# Supplementary material for: A precision medicine approach to metabolic therapy for breast cancer in mice
Source: Commun Biol. 2022 May 20;5:478. doi: 10.1038/s42003-022-03422-9 (PMC9122928; doi:10.1038/s42003-022-03422-9)
Supplement: Supplementary file 2 — Supplementary Information [file 42003_2022_3422_MOESM2_ESM.pdf]

Cell Line	Mouse Strain	Cells injected
4T1	BALB/c	$1x10^4$
Ac711	C57bl/6J	$1x10^6$
EMT6	BALB/c	$1x10^6$
Eph4 1424	BALB/c	$5x10^5$
M158	C57bl/6J	$1x10^6$
M6	FVB	$2x10^5$

Supplementary Table 1. Details of the subcutaneous tumor cell injection protocols.

Cell Line	Strain	Source	Mutation(s)
4T1	BALB/c	ATCC	p53, Pik3cg
Ac711	C57bl/6J	ATCC	HRAS
EMT6	BALB/c	ATCC	PTEN
Eph4 1424	BALB/c	ATCC	MEK1
M158	C57bl/6J	ATCC	MYC
M6	FVB	ATCC	P53
MMTV-PyMT	FVB	Jackson Labs	ERBB2, p53

Supplementary Table 2. Details of the models tested in this study.

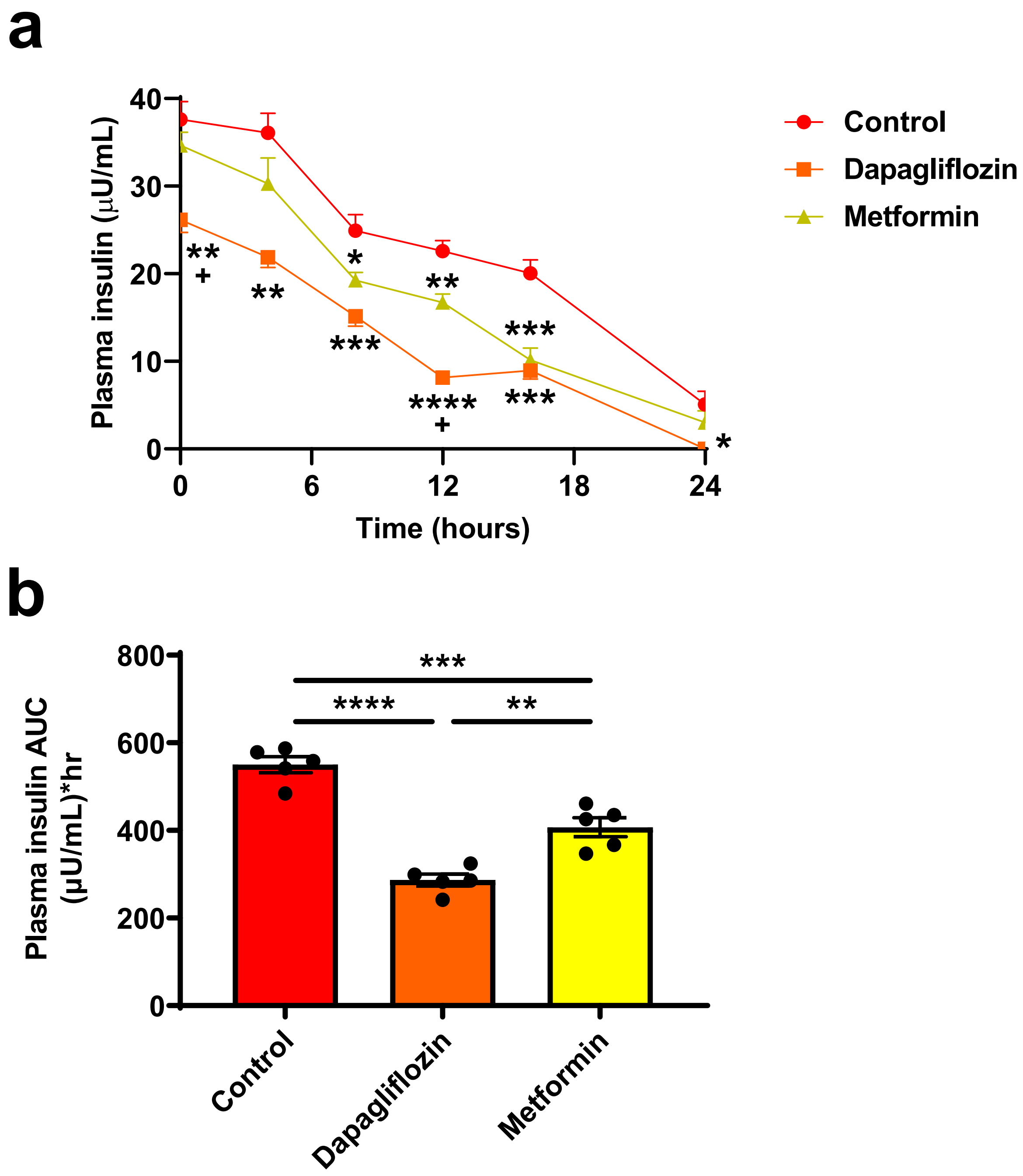

Supplementary Figure 1. In Western diet fed mice, dapagliflozin is more effective at lowering the insulin area under the curve (AUC) than metformin. (a) Plasma insulin concentrations. Drugs were administered and food was removed at time zero. Data are the mean±S.E.M. of n=5 per group. *P<0.05, **P<0.01, ***P<0.001, ****P<0.0001 vs. control; +P<0.05 vs. metformin. Groups were compared by ANOVA with Tukey's multiple comparisons test. (b) Insulin area under the curve throughout the 24 hr period. **P<0.01, ***P<0.001, ****P<0.0001 by ANOVA with Tukey's multiple comparisons test.

Acute dapagliflozin treatment

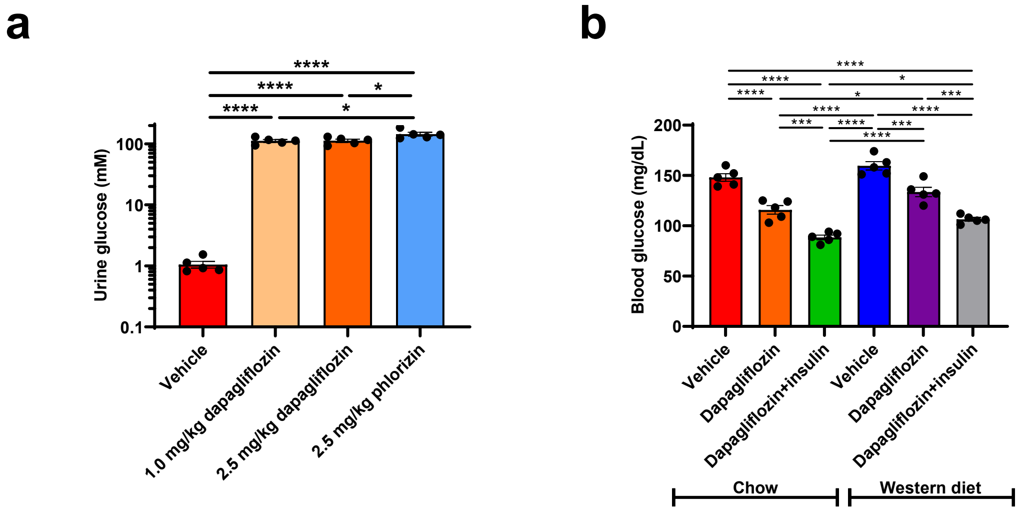

Chronic dapagliflozin treatment

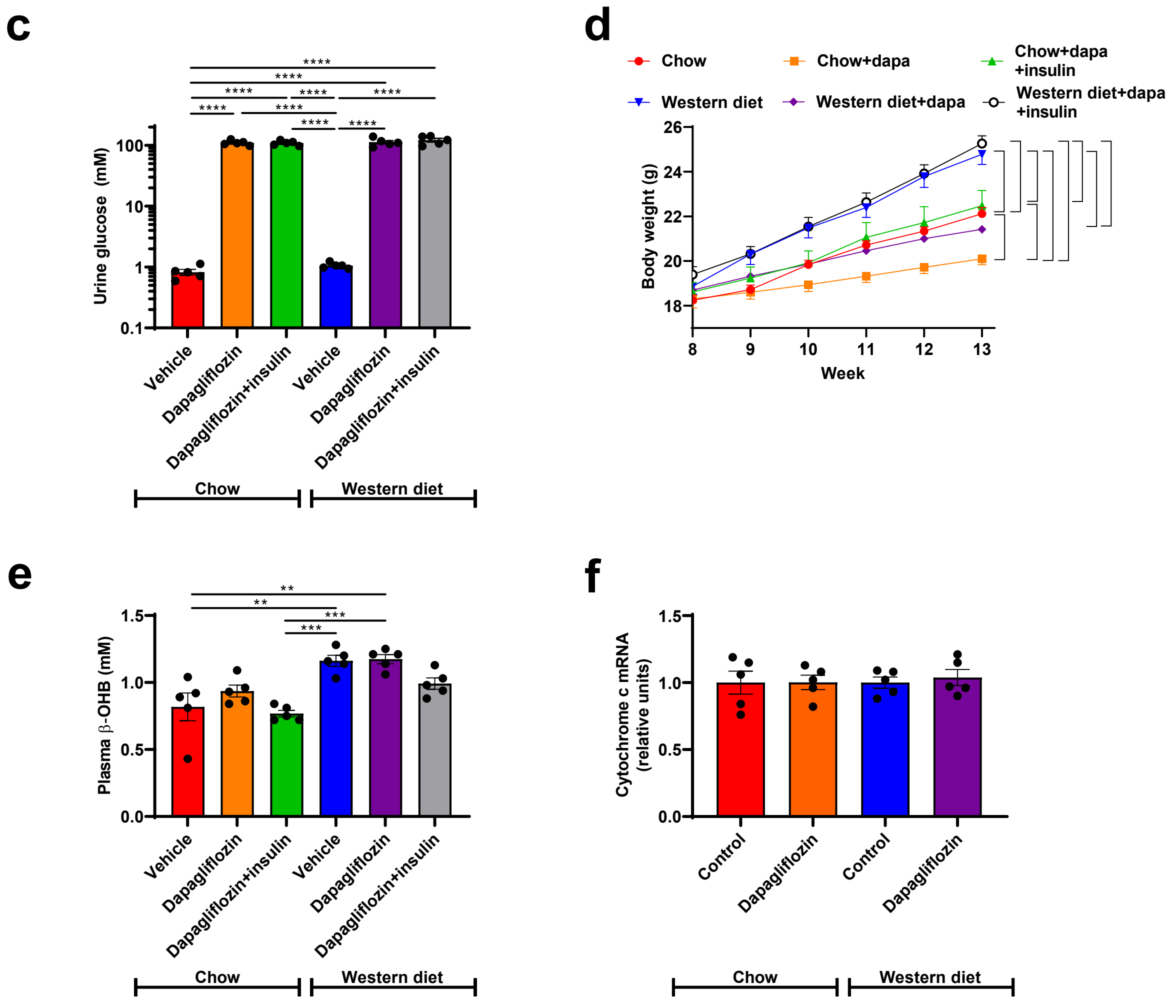

Supplementary Figure 2. Dapagliflozin slows tumor growth in both lean and obese MMTV-PyMT mice, correlated with reductions in plasma insulin. (a) Urine glucose in mice treated acutely with dapagliflozin or phlorizin. (b) Blood glucose in mice treated acutely with dapagliflozin±insulin. (c) Urine glucose and (d) Body weight in mice treated chronically (4 weeks) with dapagliflozin. Brackets indicate statistically significant (P<0.05) comparisons. The mean±S.E.M. of n=5 per group is shown. (e) Plasma β-hydroxybutyrate. (f) Tumor cytochrome c mRNA expression. In all panels, n=5 per group, and *P<0.05, **P<0.01, ***P<0.001, ****P<0.0001 by ANOVA with Tukey's multiple comparisons test.

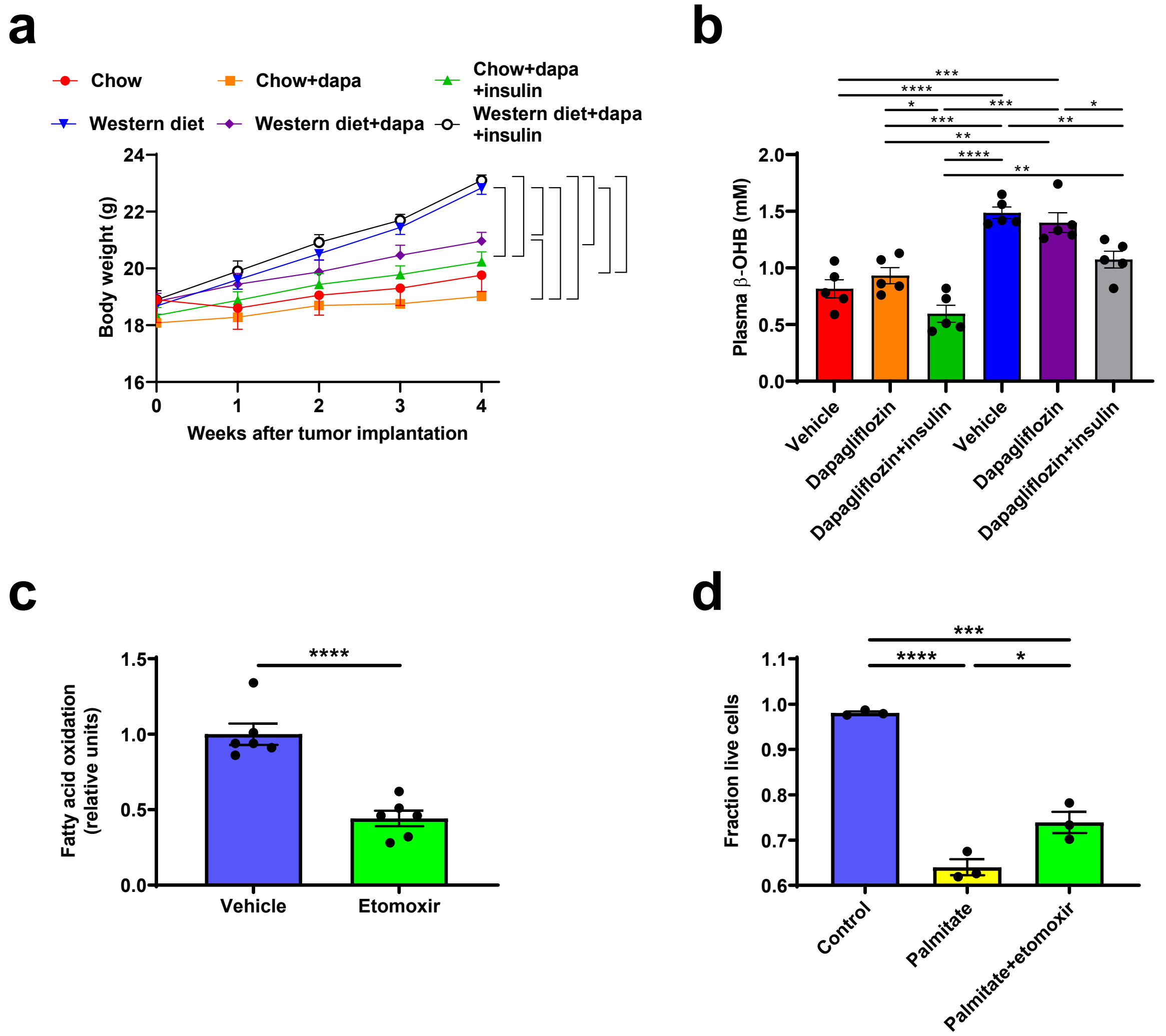

Supplementary Figure 3. Dapagliflozin slows tumor growth in both lean and obese 4T1 tumor-bearing mice, correlated with reductions in plasma insulin. (a) Body weight. Data are the mean±S.E.M. of n=5 per group. Brackets denote statistically significant (P<0.05) comparisons. (b) Plasma β-OHB. n=5 per group. (c) Validation of etomoxir: fatty acid oxidation with or without etomoxir. n=6 replicates per condition. ****P<0.0001 by the 2-tailed unpaired Student's t-test. (d) Fraction live 4T1 cells cultured in palmitate with or without etomoxir. n=3 replicates per condition. In panels (a), (b), and (d), *P<0.05, **P<0.01, ***P<0.001, ****P<0.0001 by ANOVA with Tukey's multiple comparisons test.

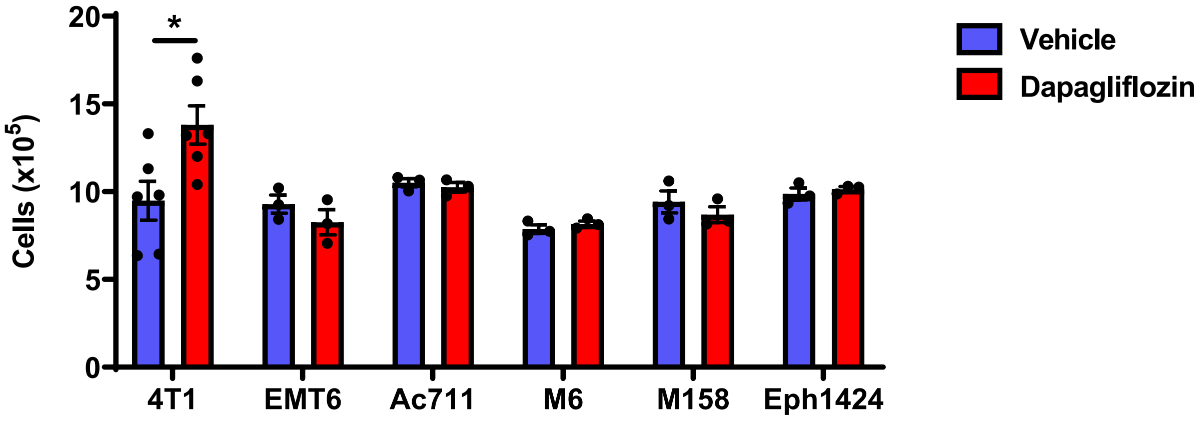

Supplementary Figure 4. Dapagliflozin does not slow tumor growth *in vitro*. Cells were cultured in vehicle (0.5% DMSO) or dapagliflozin (100 μM) for 48 hr. **P*<0.05 by the 2-tailed unpaired Student's t-test. n=6 (4T1) or 3 (all other cell lines) replicates per condition.
