# Supplementary material for: A precision medicine approach to metabolic therapy for breast cancer in mice
Source: Commun Biol. 2022 May 20;5:478. doi: 10.1038/s42003-022-03422-9 (PMC9122928; doi:10.1038/s42003-022-03422-9)
Supplement: Supplementary file 5 — Reporting Summary [file 42003_2022_3422_MOESM5_ESM.pdf]

nature portfolio

Corresponding author(s): Rachel J. Perry

Last updated by author(s): 03-28-2022

Reporting Summary

Nature Portfolio wishes to improve the reproducibility of the work that we publish. This form provides structure for consistency and transparency in reporting. For further information on Nature Portfolio policies, see our Editorial Policies and the Editorial Policy Checklist.

Statistics

For all statistical analyses, confirm that the following items are present in the figure legend, table legend, main text, or Methods section.

n/a	Confirmed	
☐	☒	The exact sample size (*n*) for each experimental group/condition, given as a discrete number and unit of measurement
☐	☒	A statement on whether measurements were taken from distinct samples or whether the same sample was measured repeatedly
☐	☒	The statistical test(s) used AND whether they are one- or two-sided<br>*Only common tests should be described solely by name; describe more complex techniques in the Methods section.*
☐	☒	A description of all covariates tested
☐	☒	A description of any assumptions or corrections, such as tests of normality and adjustment for multiple comparisons
☐	☒	A full description of the statistical parameters including central tendency (e.g. means) or other basic estimates (e.g. regression coefficient) AND variation (e.g. standard deviation) or associated estimates of uncertainty (e.g. confidence intervals)
☐	☒	For null hypothesis testing, the test statistic (e.g. *F*, *t*, *r*) with confidence intervals, effect sizes, degrees of freedom and *P* value noted<br>*Give P values as exact values whenever suitable.*
☒	☐	For Bayesian analysis, information on the choice of priors and Markov chain Monte Carlo settings
☒	☐	For hierarchical and complex designs, identification of the appropriate level for tests and full reporting of outcomes
☒	☐	Estimates of effect sizes (e.g. Cohen's *d*, Pearson's *r*), indicating how they were calculated

Our web collection on statistics for biologists contains articles on many of the points above.

Software and code

Policy information about availability of computer code

Data collection	No code was used. We include a statement that statistical analysis was performed using GraphPad Prism version 9.3.1.
Data analysis	No code was used.

For manuscripts utilizing custom algorithms or software that are central to the research but not yet described in published literature, software must be made available to editors and reviewers. We strongly encourage code deposition in a community repository (e.g. GitHub). See the Nature Portfolio guidelines for submitting code & software for further information.

Data

Policy information about availability of data

All manuscripts must include a data availability statement. This statement should provide the following information, where applicable:

- Accession codes, unique identifiers, or web links for publicly available datasets
- A description of any restrictions on data availability
- For clinical datasets or third party data, please ensure that the statement adheres to our policy

Our data availability statement is as follows: "Individual data points are shown throughout, except in line graphs where so many data points are present that it would be impossible to pick out individual data points. All raw data are available from the corresponding author upon request."

Field-specific reporting

Please select the one below that is the best fit for your research. If you are not sure, read the appropriate sections before making your selection.

☒ Life sciences ☐ Behavioural & social sciences ☐ Ecological, evolutionary & environmental sciences

For a reference copy of the document with all sections, see nature.com/documents/nr-reporting-summary-flat.pdf

Life sciences study design

All studies must disclose on these points even when the disclosure is negative.

Sample size	We have added a statement confirming that sample sizes were chosen using a power calculation to provide adequate power to detect large differences (as would be required for future clinical trial design) with moderate error.
Data exclusions	No data were excluded from analysis.
Replication	The primary means of verification of reproducibility was the fact that studies were performed in seven separate breast cancer mouse models.
Randomization	Animals were randomized to experimental groups with the use of a random number generator.
Blinding	We now state that all metabolic and tumor size analyses were formed by an investigator who was blinded as to group allocation.

Reporting for specific materials, systems and methods

We require information from authors about some types of materials, experimental systems and methods used in many studies. Here, indicate whether each material, system or method listed is relevant to your study. If you are not sure if a list item applies to your research, read the appropriate section before selecting a response.

Materials & experimental systems

n/a	Involved in the study	
☒	☐	Antibodies
☐	☒	Eukaryotic cell lines
☒	☐	Palaeontology and archaeology
☐	☒	Animals and other organisms
☒	☐	Human research participants
☒	☐	Clinical data
☒	☐	Dual use research of concern

Methods

n/a	Involved in the study	
☒	☐	ChIP-seq
☒	☐	Flow cytometry
☒	☐	MRI-based neuroimaging

Eukaryotic cell lines

Policy information about cell lines

Cell line source(s)	Each cell line was obtained from ATCC.
Authentication	Authentication was not performed in our laboratory; however, the cell lines are routinely authenticated at ATCC.
Mycoplasma contamination	All cells were confirmed pathogen free prior to injection by the Yale Comparative Pathology Research Core.
Commonly misidentified lines (See ICLAC register)	N/A

Animals and other organisms

Policy information about studies involving animals; ARRIVE guidelines recommended for reporting animal research

Laboratory animals	The use of female mice, the strains, and the ages of the mice are reported in the text.
Wild animals	The study did not involve wild animals.
Field-collected samples	The study did not involve samples collected from the field.
Ethics oversight	We state that the Yale Institutional Animal Care and Use Committee approved the protocols used in the study.

Note that full information on the approval of the study protocol must also be provided in the manuscript.
